# The impact of maternal work–family conflict on problem behaviors among preschoolers during the COVID-19 epidemic: a moderated mediation model of maternal anxiety and trait mindfulness

**DOI:** 10.3389/fpsyg.2023.1290597

**Published:** 2023-11-17

**Authors:** Xiuzhen Jin, Jinkyeong Ahn

**Affiliations:** Kunsan National University, Gunsan, North Jeolla, Republic of Korea

**Keywords:** work–family conflict, maternal anxiety, preschoolers’ problem behaviors, trait mindfulness, intermediation effect, moderating effect

## Abstract

**Purpose:**

The mediating effects of maternal anxiety and moderating effects of trait mindfulness on the relationship between Work–family conflict (WFC) and preschool children’s problem behaviors remain unclear during the COVID-19 epidemic. So, this study examined the association between mothers’ WFC and preschoolers’ problem behaviors and identified the roles of maternal anxiety as a mediator and trait mindfulness as a moderator during the COVID-19 epidemic.

**Methods:**

In this cross-sectional study, a sample of 1,068 Chinese preschoolers and their mothers from coastal cities in southern China were investigated. Data were collected using demographic questionnaires, Carlson’s WFC scale, Ma’s Parenting Anxiety Scale, Goodman’s SDQ Scale, and Brown and Ryan’s Trait Mindfulness Scale, and were analyzed using SPSS 26.0 and Process 3.3.

**Results:**

WFC had a positive and direct association with problem behaviors in preschoolers (*β* = 0.118, *t* = 3.880, *p* < 0.001). WFC also had a positive and direct association with maternal anxiety (*β* = 0.480, *t* = 18.034, *p* < 0.001). Maternal anxiety had a positive and direct association with preschoolers’ problem behaviors (*β* = 0.415, *t* = 13.584, *p* < 0.001). The mediating effect value of maternal anxiety between WFC and preschoolers’ problem behaviors was 0.199, and the moderating effect value of trait mindfulness between maternal anxiety and preschoolers’ problem behaviors was −0.078.

**Conclusion:**

WFC was positively associated with preschoolers’ problem behaviors, and maternal anxiety was a mediator of this association. So, WFC could cause maternal anxiety and lead to more problematic behaviors in children. Besides, maternal anxiety was positively associated with preschoolers’ problem behaviors, and trait mindfulness was a moderator of this association.

## Introduction

Problem behavior, known as behavior distress, bad behavior, and so on, is an important indicator of individual social adaptation. Onyskiw believes that problem behavior is a series of behaviors that are contrary to common sense standards in the process of children’s socialization (Onyskiw, 2003), including the problem behaviors of emotion, attention deficit, conduct, and peer relationship (Durant et al., 1997). While Achenbach used a dichotomy to divide problem behaviors into internalized problem behaviors and externalized problem behaviors (Achenbach, 1991). Internalized problem behaviors refer to the negative emotions that individuals experience physiologically, including anxiety, depression, withdrawal, and other emotional problems (Zahn-Waxler et al., 2000; Onyskiw, 2003). Externalized problem behaviors refer to non-adaptive behaviors that violate social norms (Liu, 2004), including disciplinary and aggressive behaviors. Numerous studies have shown that both internalized and externalized problem behaviors are distributed in the preschool period. In recent years, children’s problem behaviors have been increasingly younger (Chen et al., 2021). Due to the rapid physical and mental development of individuals in the preschool period, and at the same time individuals experience changes in the environment from home to school, it is easier for preschoolers to produce problem behaviors (Peng et al., 2018). The emergence of problem behaviors will directly affect preschoolers learning knowledge and skills. Besides, problem behaviors in early childhood will continue into adolescence if not intervened, eventually leading to an increase in the probability of adverse behaviors such as substance abuse in adolescence and even adulthood (Ashford et al., 2008; Fanti and Henrich, 2010; Basten et al., 2016). Rutter’s early research found that more than 70% of the subjects with antisocial problem behaviors in adulthood had a strong tendency toward antisocial behaviors in childhood (Robins and Price, 1991). Therefore, problem behaviors in early childhood have a great impact on individual growth.

### Work–family conflict and preschoolers’ problem behaviors

Work–family conflict refers to the role conflict that occurs when an individual’s family needs and work needs are difficult to coordinate (Li et al., 2017). This kind of role conflict, involving both home and work, is relevant to every professional worker, especially mothers who take full-time jobs. The dual identity of a professional worker and the mother of one or more minor children often faces greater role conflict (Zhou et al., 2018), which may have been amplified by COVID-19. Since the outbreak of COVID-19, many countries have implemented social distancing measures to curb its spread, which has also fundamentally changed mothers’ routines in the home and work spheres (Prime et al., 2020; Settersten et al., 2020). Many mothers are required to take full responsibility for the supervision, care, and education of their children during working hours as a result of the measures that have resulted in a significant increase in the time children spend with their mothers. So, a significant proportion of mothers will bring work into the family (Arntz et al., 2020), which also creates new risks for parent–child relationships and child development (Adisa et al., 2021; Schmeer et al., 2023). Previous studies have focused on the impact of work–family conflict on individual work or psychological aspects (such as job burnout, life satisfaction, etc.), but little is known about the impact of work–family conflict on child development (Dinh et al., 2017). Therefore, this study attempts to change the perspective and explore the impact of work–family conflict on child development.

Research has found that mothers working from home blur the boundaries between work and parenting roles (Desrochers and Sargent, 2004) and that mothers managing these boundaries deplete their psychological resources and negatively impact their parenting behaviors (Voydanoff, 2005). Meanwhile, as these boundaries become more permeable (Desrochers and Sargent, 2004), this also leads to more tasks, longer working hours, more work–family conflict, and increased stress, especially when they spend overtime working (Arntz et al., 2019; Kim et al., 2020; Song and Gao, 2020). According to the spillover hypothesis theory, individual emotions (including positive and negative) or behaviors will be transferred from one situation (relationship) to another situation (relationship). Work–family conflict, as a negative emotional perception, may be transferred to the parenting situation and have a negative impact on children. Studies have shown that when mothers have conflicts between work and family, children are more likely to have externalized problem behaviors, such as emotional problems (e.g., depression and anxiety), and behavioral problems (e.g., hyperactivity) (Zeng, 2011; Hess and Pollmann-Schult, 2020; Wang et al., 2021). For example, Strazdins found that when the mother experienced a high level of work–family conflict, the emotional and behavioral problems of 4 or 5-year-old children would be more prominent, and this association has a certain stability (Strazdins et al., 2013). As a result, mothers working from home are likely to amplify the negative spillovers of work-related stress to parent–child relationships, which in turn leads to more problem behaviors in children (Edhborg et al., 2003). Before the epidemic, some scholars have already confirmed that mothers’ work–family conflict can significantly predict children’s anxiety and other internalized problem behaviors (Zeng, 2011). However, the mechanistic research on the relationship between mothers’ work–family conflict and preschoolers’ problem behaviors caused by the COVID-19 epidemic was still very limited. Therefore, this study explored the association between work–family conflict and preschoolers’ problem behaviors during the COVID-19 epidemic, and the underlying mechanisms of maternal anxiety and trait mindfulness in the Chinese context.

### Maternal anxiety as a mediator

Research has found that the COVID-19 epidemic has increased work–family conflict for many mothers (Verweij et al., 2021). And both pre-pandemic and during-pandemic studies have shown that work–family conflict is associated with higher levels of anxiety (Freisthler et al., 2021) and health problems (Borgmann et al., 2019), and high levels of work–family conflict often lead to lower maternal sensitivity (Chung and Van der Lippe, 2020; Haines III et al., 2020), which creates a greater risk of increased problem behaviors in children (Conrad and Hammen, 1989). Moreover, the work–family conflict theory emphasizes that in the case of lack of boundary elasticity, role stress, and role conflict will cause individuals to have more negative emotional experiences such as anxiety (Greenhaus and Beutell, 1985; Eby et al., 2005). In the Chinese social environment, the work–family conflict also has a significant impact on mothers’ parenting anxiety (Zhang and Lin, 2020), individual work anxiety (Qiu et al., 2017), and mental health (Zeng et al., 2019). Staines proposed the spillover theory of the relationship between work and family in 1980. This theory holds that work–family conflict will have adverse effects on both work and family, while work–family conflict, as a stressor, can extend the negative impact of mothers’ negative emotional level on parent–child relationships, resulting in maladaptive problems in children (Cox and Paley, 1997; Buehler and Gerard, 2002). It can even cause distress and anxiety in children, leading to a series of problem behaviors (Su et al., 2011). According to social learning theory, mothers’ negative emotions (such as anxiety) can affect the child’s problem behaviors level through the role of genetics and parenting style (Puka et al., 2017).

A survey study using a family research design, after controlling for the influence of parental raters, also showed that maternal anxiety symptoms significantly predicted children’s problem behaviors (Gagne et al., 2019). Meanwhile, a large cohort study conducted in Norway using multilevel analysis modeling found that maternal anxiety was significantly associated with internalized problems in preschoolers (Gjerde et al., 2020). The findings of Teyhan suggested that maternal anxiety symptoms were significantly associated with children’s internalized problems and abnormal SDQ difficulty total scores (Teyhan et al., 2014). In addition, a large-scale cross-sectional study conducted by Chinese scholars found that during the COVID-19 epidemic, maternal anxiety had a greater impact on children, that is, the higher the anxiety level of parents, the higher the incidence of children’s problem behaviors (Zhong et al., 2021). And a long-term, multi-measurement follow-up study conducted by Frigerio before and after the COVID-19 epidemic showed that in a sample of preschoolers, the more severe the mother’s emotional symptoms, the higher incidence of children’s internalized (i.e., emotional responses, anxiety/depression, withdrawal) and externalized (i.e., aggressive behaviors) problems. This also suggests that in stressful settings (such as those created by the COVID-19 epidemic and subsequent illness), mothers’ emotional symptoms may have some effects on the child’s healthy development negative effects (Frigerio et al., 2022). And the results of a follow-up study conducted during the COVID-19 epidemic in Israel also supported a significant association between maternal anxiety and children’s internalized problems (Hanetz-Gamliel et al., 2021). Therefore, this study hypothesizes that maternal anxiety acts as the mediator between maternal work–family conflict and preschoolers’ problem behaviors during the COVID-19 epidemic.

### Maternal trait mindfulness as a moderator

Ellen Langer believes that mindfulness is a psychological trait and it refers to the degree to which individuals perceive and pay attention to stimuli that occur at the moment, with stability across time and situations (Brown and Ryan, 2003). The mindful coping model holds that individuals with high mindfulness can expand attention and enhance cognitive flexibility when they decentralize their responses to conflicts and other potential threats in the environment, and carry out positive cognitive reappraisal, thus reducing the threat brought by conflicts or stressful events (Garland et al., 2009). Therefore, mothers facing high levels of work–family conflict do not necessarily experience negative effects due to the role of individual mindfulness. The results of empirical research also showed that maternal mindfulness had a positive effect on reducing the negative impact of conflict and problem behaviors of children (Siu et al., 2016). Besides, an empirical study in the Chinese context suggested that maternal mindfulness effectively alleviated the impact of stress caused by individual work–family conflict on children’s problem behaviors (Wang et al., 2023). And the family interaction theory holds that the interaction between family members and the interaction model between parents and children can effectively explain the formation mechanism of children’s problem behaviors (Lv et al., 2003). For preschoolers, mothers are the primary caregivers and significant persons, so young children’s problem behaviors are inevitably influenced by some of the mother’s traits, such as mindfulness (Goodman et al., 2011; Corthorn, 2018). Studies have found that higher levels of mindfulness in mothers tend to predict fewer problem behaviors and positive social adaptation in children (Geurtzen et al., 2015; Coatsworth et al., 2018). And Siu found that maternal mindfulness levels were negatively correlated with children’s problem behaviors, and high levels of maternal mindfulness helped to improve young children’s problem behaviors (Siu et al., 2016). Therefore, this study hypothesizes that maternal trait mindfulness moderates the direct link between work–family conflict and problem behaviors in preschoolers during the COVID-19 pandemic.

Research has shown that individuals with trait mindfulness are good at mastering their attention and making non-judgment about what is happening at the moment, which helps to regulate the individual’s emotional state (Gross, 1998; Shallcross and Spruill, 2018). Individuals with high trait mindfulness can look at things dialectically, quickly get rid of the negative emotions brought about by negative events (Haun et al., 2018), and are less likely to be influenced by the outside world (Xu et al., 2017). On the contrary, individuals with low trait mindfulness have difficulty focusing on the present moment, and stressful events in daily work and life can trigger more severe mood swings (Xu et al., 2015). The study by Wei Xu also found that trait mindfulness had a moderating effect on the relationship between stress and individual emotions (Xu et al., 2017). For individuals with high trait mindfulness, stress is a weaker positive predictor of their mood. So trait mindfulness plays a mediating role between stressful events and individual emotions. Therefore, when faced with stressful events such as the COVID-19 epidemic, maternal trait mindfulness may be able to alleviate the adverse relationship between work–family conflict and mothers’ anxiety.

In addition, many scholars in recent years have introduced individual mindfulness into the field of family parenting, which is associated with child development outcomes. The advancement of numerous studies has led to the gradual rise of the Mindfulness-Based Intervention Program (MBI) for mothers. Intervention research results showed that a period of mindfulness intervention for mothers effectively reduced mothers’ own stress and parenting pressure, improved anxiety and other emotional problems and negative parenting behaviors, and reduced children’s problem behaviors (Coatsworth et al., 2018; Chaplin et al., 2021). Besides, family system theory believes that the various family factors that affect children’s development do not work independently, but also have interactive joint effects (Cox and Paley, 1997), that is, as an individual factor of the mother in the family system, maternal trait mindfulness can interact with the mother’s anxiety and work–family conflict to some extent, thus affecting the development of children. Research confirmed that mothers’ mindfulness not only affected their own physical and mental health but also migrated to their parenting behaviors, thus indirectly affecting child development (Gouveia et al., 2016; Corthorn, 2018; Han et al., 2021). Mothers with a higher level of mindfulness can perceive and pay attention to the current experience in life and have strong self-compassion (Pepping et al., 2013). In addition, in parent–child interaction, they are often well aware of negative emotional experiences, effectively regulate and improve their emotional states, better perceive children’s feelings, promote children’s healthy development, and reduce the risk of children’s problem behaviors (Moreira and Canavarro, 2017; Benton et al., 2019). Thus, maternal trait mindfulness may also moderate the association between maternal anxiety and problem behaviors in preschoolers.

### The present study

**Figure 1:**
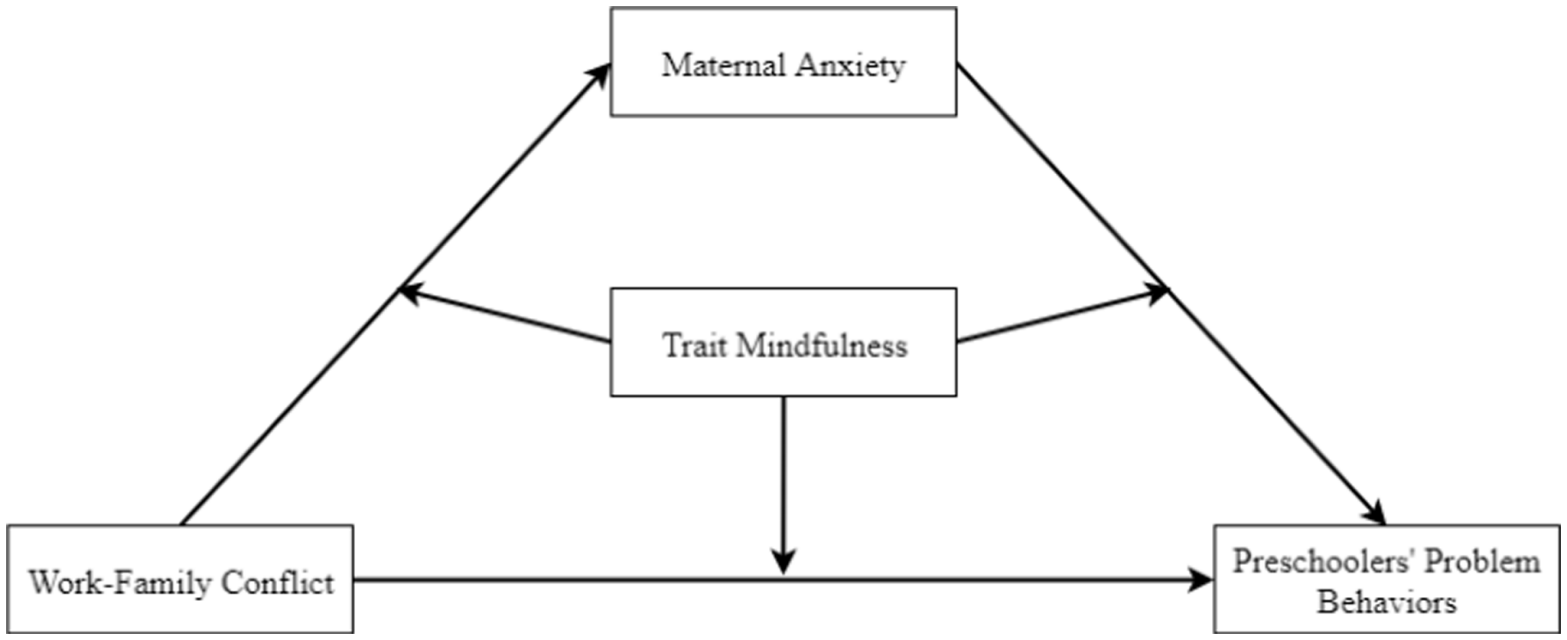
The conceptual moderated mediation model of work–family conflict on preschoolers’ problem behaviors during the COVID-19 epidemic.

Based on the spillover theory (Staines, 1980), social learning theory (Puka et al., 2017), and family systems theory (Cox and Paley, 1997), the present study tested a moderated mediation model to clarify the mechanisms underlying the associations between work–family conflict and preschoolers’ problem behaviors during the COVID-19 epidemic. This integrated model indicated how and when work–family conflict influenced preschoolers’ problem behaviors during the COVID-19 epidemic (Figure 1). Based on the literature review, the following hypothesizes were generated:

*H1*: Maternal work-family conflict is significantly associated with preschoolers’ problem behaviors.

*H2:* Maternal anxiety mediates the association between work-family conflict and preschoolers’ problem behaviors.

*H3*: Direct and/or indirect associations between work-family conflict and preschoolers’ problem behaviors due to maternal anxiety varied by maternal trait mindfulness engagement.

## Method

### Participants and procedure

We conducted the study in June 2022. Due to the import of overseas infection cases and other reasons, the situation of the COVID-19 epidemic in China was still severe, during which parents and their children were both at home and mothers spent significantly more time with their children because of the government’s isolation policy. Therefore, there was an unprecedented close connection between parents and their children during the COVID-19 epidemic. Then, we used a cross-sectional online questionnaire to assess the relationship between work–family conflict, maternal anxiety, trait mindfulness, and preschoolers’ problem behaviors during the COVID-19 epidemic. And, the Research Ethics Committee of our University approved the study.

First, mothers were given a link that opened an online consent form describing their rights, which informed them that the data would be used only for research purposes, participation was voluntary, and refusal to participate and withdrawal from the study would not lead to negative consequences. Then, mothers decided whether they consented to their own and their children’s participation in this study. Only if the mothers and their children agreed to participate was the online survey made available. For those mothers and their children who declined to participate, the survey ended. For all other mothers and their children, the survey began with questions regarding demographics (e.g., age), followed by questions on work–family conflict, maternal anxiety, trait mindfulness, and preschoolers’ problem behaviors.

Finally, the current study recruited 1,116 mothers and their children via convenient cluster sampling from a city in Southeast China; 48 parents and their children refused to participate and withdrew from the study, for a non-response rate was 4.30% (48/1116). Invalid samples included subjects with response regularity and missing values in the data. All valid samples have completed all questions, and no missing values exist. The final effective sample included 1,068 mothers and their children. Of the children, 39, 424, 369, and 236 were in the infant, small, middle, and large classes, respectively. Child participants were aged between 2.5 and 6.8 years (*M* = 4.50, *SD =* 0.84 years; 49.81% boys). And in the mother samples, the participants were aged between 23 and 48 years (*M* = 38.09, *SD =* 8.92 years).

### Measures

#### Work-family-conflict

The Work–Family Conflict Scale was developed by Carlson (Carlson et al., 2000), was introduced to China by scholars in 2007 and its effectiveness was verified (Gan, 2007). The scale consists of 18 items divided among the dimensions of work-family and family-work. And each dimension is divided into three levels: time-based, behavior-based, and stress-based. Items are answered on a five-point Likert scale ranging from 1 (totally disagree) to 5 (totally agree). In this study, higher scores indicated higher levels of work–family conflict faced by mothers during the COVID-19 epidemic. Cronbach’s α was 0.954 in the current study.

#### Maternal anxiety

The Parenting Anxiety Scale was developed by Ma (2013). The scale is divided into two versions. The parent version of the questionnaire was used in this study. The scale consists of 16 items divided among the dimensions of parenting awareness, parenting behaviors, parenting support, and parenting influence. Among them, there are five items each on parenting awareness and parenting behaviors, and three items each on parenting support and parenting influence. Items are answered on a five-point Likert scale (1 = never or almost no to 5 = every day or almost every day). The scale was rated by the mother, with higher scores indicating higher levels of maternal anxiety. In the current study, Cronbach’s α for this questionnaire was 0.804.

#### Preschoolers’ problem behaviors

The Strengths and Difficulties Questionnaire (SDQ) was developed by Goodman (Durant et al., 1997). The test version of this study is the parent version revised by Chinese scholars, and its validity has been verified (Kou et al., 2005). There are 25 items in total, including four difficulty dimensions (20 items), emotional problems, conduct problems, lack of attention, and peer relationship problems, and the dimension of prosocial behavior strengths (5 items). In this study, four difficulty dimensions were selected to measure the problem behaviors of preschool children, and mothers judged them by using a two-point scale ranging from 0 = very inconsistent to 2 = very consistent according to the daily performance of the preschool children in the past six months. If the score is higher than 17, the child is considered to have problem behaviors. In the current study, Cronbach’s α for this questionnaire was 0.801.

#### Maternal trait mindfulness

The Mindful Attention Awareness Scale (MAAS) was developed by Brown and Ryan (Brown and Ryan, 2003), and it was later translated into Chinese scale by Chinese scholars and tested and optimized (Chen et al., 2012). The scale consists of 15 items (e.g., “ *I could be experiencing some emotion and not be conscious of it until sometime later* ”). Items are answered on a five-point Likert scale (1 = almost always to 5 = almost never). All entries are reverse-scored. This study adopted the method of self-rating of mothers, with higher scores indicating greater trait mindfulness. In the current study, Cronbach’s α for this questionnaire was 0.920.

#### Covariates

Previous studies have shown that maternal anxiety is influenced by demographic characteristics, such as age and family socioeconomic status (SES) (Nelson, 2010; Nomaguchi and Brown, 2011; Xiang et al., 2014; Kebriyaei et al., 2020). And Preschoolers’ problem behaviors also are influenced by demographic characteristics, such as gender, age, and family socioeconomic status (SES) (Aebi et al., 2014; Flouri et al., 2014; Hosokawa and Katsura, 2018; Yu et al., 2020). These background variables were used as covariates in the analyses; gender was coded as 0 = girl, 1 = boy. For family SES, we collected household income, parents’ education, and parents’ occupations. A principal components analysis was performed, with family SES computed using the following formula: Family SES = (β1 × Z income + β2 × Z education + β3 × Z occupation) / εf, where β1–3 are the factor loadings and εf is the eigenvalue for the first factor (Cheng et al., 2021). In the current study, the participants’ family SES range was −1.656 to 2.222.

#### Analytic plan

First, descriptive statistics and correlations were obtained. Second, we examined the mediation effect of maternal anxiety. Third, we further examined whether the mediation process was moderated by maternal trait mindfulness. The analysis of moderated mediation models was performed using Hayes’s (Hayes and Scharkow, 2013) PROCESS macro (Models 4 and 59). In all analyses, we included preschoolers’ gender, age, maternal age, and family SES as control variables.

## Results

### Common method deviation test

The data in this study were all from the mother’s report. To control for the common method biases in this study, Harman’s single-factor test was conducted (Podsakoff et al., 2003). The results indicated that altogether fourteen factors had an Eigenvalue of more than 1 and could jointly explain 25.08% of the variance, which is less than the critical value (40%). Therefore, there were no significant common method biases in this study.

### Descriptive analysis

The means, standard deviations, and Pearson’s correlation coefficients for all variables were shown in Table 1. Work–family conflict and maternal anxiety were significantly positively associated with preschoolers’ problem behaviors, and the three variables were significantly negatively associated with maternal trait mindfulness.

**Table 1:** Means, standard deviations, and correlations of the main study variables.

	*M ± SD*	1	2	3	4	5	6	7	8
1. Gender of children	–	–							
2. Pediatric age	4.50 *±* 0.84	−0.017	–						
3. Maternal age	36.42 *±* 4.97	0.027	0.079**	–					
4.Family SES	−1.66 *±* 2.22	−0.015	−0.010	−0.001	–				
5. Work–family conflict	45.55 *±* 15.06	0.040	0.023	0.033	−0.102**	–			
6. Maternal anxiety	38.09 *±* 8.92	0.068*	0.052	−0.050	−0.139***	0.490***	–		
7. Preschoolers’ problem behaviors	8.95 *±* 4.47	0.111***	0.032	−0.106**	−0.113***	0.326***	0.489***	-	
8. Trait mindfulness	58.12 *±* 10.45	−0.036	−0.044	0.025	0.078*	−0.595***	−0.522***	−0.378***	–

*N* = 1,068; gender of children was dummy coded (0 = male, 1 = female); *p < 0.05, **p < 0.01, ***p < 0.001.

### Testing for mediation effect

To test Hypothesis 1 and Hypothesis 2, we adopted the steps recommended by predecessors to test the mediation effect (Wen and Ye, 2014a), and used regression analyses in turn, as shown in Table 2. Equation 1 suggested that work–family conflict had a significant impact on problem behaviors in preschoolers (*β* = 0.317, *p* < 0.001). Equation 2 suggested that the effect of work–family conflict on maternal anxiety was significant (*β* = 0.480, *p* < 0.001). And Equation 3 suggested that included in the regression equation, both work–family conflict and maternal could significantly positively predict preschoolers’ problem behaviors (*β* = 0.118, *p* < 0.001; *β* = 0.415, *p* < 0.001), which demonstrated that maternal anxiety mediated the association between work–family conflict and preschoolers’ problem behaviors.

**Table 2:** Testing the mediating effect of work–family conflict on preschoolers’ problem behaviors.

Predictors	Equation 1		Equation 2		Equation 3	
	Dependent: PPB		Dependent: MA		Dependent: PPB	
	*β*	*t*	*β*	*t*	*β*	*t*
WFC	0.317	11.036***	0.480	18.034***	0.118	3.880***
MA					0.415	13.584***
R^2^	0.137		0.257		0.265	
*F*	33.763***		73.462***		63.754***	

*N* = 1,068. The beta values are standardized coefficients. WFC, work–family conflict; PPB, preschoolers’ problem behaviors; MA, maternal anxiety. *p < 0.05, **p < 0.01, ***p < 0.001.

In this study, we employed Hayes’s (Hayes, 2017) PROCESS macro for SPSS (Model 4) to test this moderated mediation model. The mediating effect value was 0.199, and the 95% confidence interval was (0.162, 0.240), excluding 0, as shown in Table 3; the direct effect value after controlling for mediating variables was 0.118 ( *p* < 0.001). And results showed that maternal anxiety had a significant relationship between work–family conflict and preschoolers’ problem behaviors, with a ratio of 0.199/ (0.199 + 0.118) accounting for 62.90% of the total effect.

**Table 3:** Effects and bootstrapping results with the path.

Path	Indirect effect	Bootstrap standard errors	95% CI	
			Lower	Upper
WFC to PPB through MA	0.1992	0.0197	0.1617	0.2400

*N* = 1,068. WFC, work–family conflict; PPB, preschoolers’ problem behaviors; MA, maternal anxiety.

### Testing for moderated mediation

To test Hypothesis 1 and Hypothesis 2, we adopted the steps recommended by predecessors to test the mediation effect (Wen and Ye, 2014b), and used regression analyses in turn, as shown in Table 4. Equation 1 suggested that when trait mindfulness was not included as a moderator in the regression equation, work–family conflict had a significant effect on preschoolers’ problem behaviors ( *β* = 0.156, *p* < 0.001), and this effect was not mediated by maternal trait mindfulness ( *p* > 0.05). After incorporating trait mindfulness as a moderator into the regression equation, Equation 2 showed that work–family conflict had a significant effect on maternal anxiety ( *β* = 0.269, *p* < 0.001), and this effect was not mediated by maternal trait mindfulness ( *p* > 0.05). Equation 3 revealed that the effect of work–family conflict on preschoolers’ problem behaviors was not significant ( *p* > 0.05), but maternal anxiety had a significant main effect on preschoolers’ problem behaviors ( *β* = 0.360, *p* < 0.001), and more importantly, maternal trait mindfulness significantly moderated this relationship ( *β* = −0.078, *p* < 0.01). The above results suggested that trait mindfulness could moderate the relationship between maternal anxiety and preschoolers’ problem behaviors.

**Table 4:** Testing the moderated mediation effects of work–family conflict on preschoolers’ problem behaviors.

Predictors	Equation 1		Equation 2		Equation 3	
	Dependent: PPB		Dependent: MA		Dependent: PPB	
	*β*	*t*	*β*	*t*	*β*	*t*
WFC	0.156	4.459***	0.269	8.529***	0.059	1.749
TM	−0.271	−7.721***	−0.354	−11.158***	−0.138	−3.962***
WFC × TM	−0.006	−0.240	0.023	1.027	0.027	0.954
MA					0.360	11.150***
MA × TM					−0.078	−2.850**
R^2^	0.185		0.336		0.283	
*F*	34.348***		76.459***		46.291***	

*N* = 1,068. The beta values are standardized coefficients. WFC, work–family conflict; PPB: preschoolers’ problem behaviors; MA, maternal anxiety; TM, trait mindfulness. **p < 0.01, ***p < 0.001.

**Figure 2:**
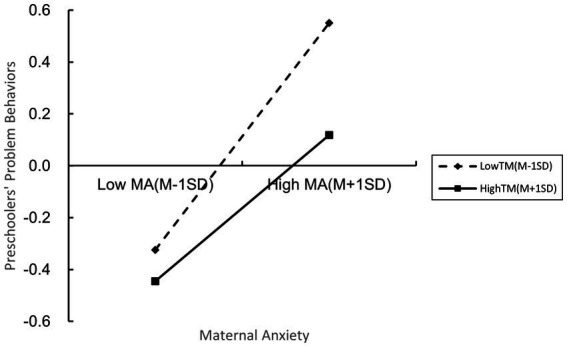
Maternal trait mindfulness as a moderator of the relationship between maternal anxiety and preschoolers’ problem behaviors during the COVID-19 epidemic.

For descriptive purposes, we plotted the relationship between maternal anxiety predicting problem behaviors in preschoolers with low and high levels of maternal trait mindfulness engagement (1 SD below the mean and 1 SD above the mean, respectively; Figure 2). From Table 5 and Figure 2, it revealed that for the group of mothers with high levels of anxiety, a high level of mindfulness of mothers would help improve the problem behaviors of children.

**Table 5:** Conditional indirect effects of maternal anxiety on Preschoolers’ problem behaviors.

	Effect	Boot SE	Boot LLCI	Boot ULCI
Low TM	0.438***	0.039	0.361	0.515
Average TM	0.360***	0.032	0.296	0.423
High TM	0.282***	0.045	0.193	0.370

***p < 0.001. *N* = 1,068. Bootstrap sample size = 5,000. LL, low limit; CI, confidence interval; UL, upper limit; Boot, bootstrap. Poor, mean, and great levels of teacher support depict 1 SD below the mean (low), mean value (average), and 1 SD above the mean (high).

## Discussion

As expected, the current study found a significant relationship between work–family conflict and preschoolers’ problem behaviors during the COVID-19 epidemic, which is similar to previous studies, which supported Hypothesis 1 (Edhborg et al., 2003; Adisa et al., 2021; Schmeer et al., 2023). The main findings elucidated the psychological mechanisms via which work–family conflict was associated with preschoolers’ problem behaviors by maternal anxiety as a mediator and maternal trait mindfulness as a moderator. The results indicated that the effect of work–family conflict on preschoolers’ problem behaviors occurred via maternal anxiety. Moreover, the relationship between work–family conflict and preschoolers’ problem behaviors via maternal anxiety was moderated by maternal trait mindfulness, which indicated that maternal trait mindfulness provided an important protective role against preschoolers’ problem behaviors during the COVID-19 epidemic.

Maternal anxiety mediated the association between work–family conflict and preschoolers’ problem behaviors, thereby supporting Hypothesis 2. In the current study, we extracted work–family conflict associated with the COVID-19 epidemic as a risk factor and maternal anxiety as an intermediary factor (Cox and Paley, 1997; Buehler and Gerard, 2002). The results supported the work–family conflict theory (Greenhaus and Beutell, 1985; Eby et al., 2005)， and proved that when individuals faced high-level work–family conflict, more anxiety would be generated, the same with the research results of Chinese scholars on mothers’ parenting anxiety (Zhang and Lin, 2020). After further analysis, this result also supported the spillover theory (Cox and Paley, 1997; Buehler and Gerard, 2002). Higher levels of maternal anxiety associated with conflict were associated with more problem behaviors in children, and the results of this study were supported by numerous empirical studies conducted before and after the COVID-19 pandemic (Hanetz-Gamliel et al., 2021; Zhong et al., 2021; Frigerio et al., 2022). The results demonstrated that both work–family conflict (risk factor) and maternal anxiety (intermediary factor) were important predictors of preschoolers’ problem behaviors, and maternal anxiety (intermediary factor) played an important mediating role in the association between work–family conflict (risk factor) and preschoolers’ problem behaviors.

A higher level of work–family conflict is associated with more maternal anxiety (Borgmann et al., 2019) and thereby with more problem behaviors in preschoolers (Cox and Paley, 1997; Buehler and Gerard, 2002). As a challenge brought to many families, the COVID-19 epidemic has led to a significant increase in more mothers working at home (Arntz et al., 2020; Prime et al., 2020; Settersten et al., 2020), and it is also closely related to the physical and mental health of mothers and their children. Thus, COVID-19 and its countermeasures are more stressful current events for parent–child groups, including mothers working from home and their preschool children, which increases maternal anxiety. The results of this study showed that when the mother had a higher level of anxiety, it would have a certain negative impact on the development of children and would make children appear more problem behaviors.

### The moderated mediation roles of maternal anxiety and trait mindfulness

The main objective of the study was to explore the moderating effect of maternal trait mindfulness on the direct link between work–family conflict and preschoolers’ problem behaviors, and the indirect link via maternal anxiety. First, the current study found that maternal anxiety was significantly, negatively, and directly associated with work–family conflict and preschoolers’ problem behaviors. Consistent with previous studies, maternal trait mindfulness was an important protective factor against their own negative emotions (Gross, 1998; Haun et al., 2018; Shallcross and Spruill, 2018) and reduced preschoolers’ problem behaviors (Moreira and Canavarro, 2017; Benton et al., 2019). Interventions that raise the level of mindfulness in mothers can effectively improve the individual’s anxiety and reduce preschoolers’ problem behaviors (Coatsworth et al., 2018; Chaplin et al., 2021). In addition, the results indicated that for mothers with high levels of anxiety, high levels of mindfulness of mothers would help improve children’s problem behaviors, and this conclusion was also supported by previous empirical studies (Gross, 1998; Moreira and Canavarro, 2017; Haun et al., 2018; Shallcross and Spruill, 2018; Benton et al., 2019). Maternal trait mindfulness did not moderate the direct link between work–family conflict and preschoolers’ problem behaviors, and did not moderate the link between work–family conflict and maternal anxiety; only the interaction between maternal anxiety and trait mindfulness significantly predicted preschoolers’ problem behaviors, which partly supported Hypothesis 3. Mothers with low trait mindfulness showed stronger associations between maternal anxiety and preschoolers’ problem behaviors than mothers with high trait mindfulness, suggesting that the association between maternal anxiety and preschoolers’ problem behaviors increases progressively with decreasing levels of maternal trait mindfulness. As a protective factor, maternal trait mindfulness can help mothers with high anxiety improve their children’s problem behaviors. The results also supported the family systems theory (Cox and Paley, 1997). Based on the family systems theory and the reality of the COVID-19 epidemic, we considered maternal anxiety as a family risk factor (Teyhan et al., 2014). The results of the moderated mediation model showed that trait mindfulness played a significant moderating role in the latter half of the mediating process. Therefore, subsequent intervention studies could attach importance to the role of maternal mindfulness level as a protective factor.

In addition, a fair amount of maternal anxiety may lead to problem behavior in preschoolers (Shamir-Essakow et al., 2005; Bögels and Brechman-Toussaint, 2006; Nicol-Harper et al., 2007). Combining family system theory with the empirical evidence above, it is indicated that there existed protective factors that moderate the relationship between maternal anxiety and preschoolers’ problem behaviors (Lv et al., 2003). As mothers are the primary caregivers and significant persons of their children, young children’s problem behaviors are inevitably influenced by some of the mother’s traits, such as mindfulness (Goodman et al., 2011; Corthorn, 2018). And the moderating role of maternal trait mindfulness was similar to that reported in previous similar studies, as studies have found that maternal mindfulness indirectly affects children’s problem behaviors through mindful parenting and positive and negative parenting behaviors (Han et al., 2021). At the same time, due to age, preschoolers’ coping methods in the face of negative events are not mature (Vu et al., 2016); accordingly, when they are exposed to maternal negative emotions for a long time during the COVID-19 epidemic, they may perform problem behaviors such as anxiety similar to their mothers (Elgar et al., 2004). And living with anxious mothers for a long time will also have adverse effects on children’s social interaction and academic performance (Downey and Coyne, 1990). After a long period of COVID-19 that has rebounded several times, mothers inevitably have a lot of negative emotions when facing the dual pressures of work and family. During the COVID-19 epidemic, while preschoolers were studying at home, mothers were also working from home, which not only resulted in more interactions between mothers and children than usual but also resulted in a more profound impact on children in all aspects. Therefore, it is urgent to alleviate negative emotions such as maternal anxiety and other negative emotions on children’s development by improving the level of maternal mindfulness.

### Practical implications

This study has important implications for preventing preschoolers’ problem behaviors during the COVID-19 epidemic. The work–family conflict caused by COVID-19 presents a major challenge to the mental health of mothers and the healthy development of preschoolers, especially anxiety and other negative emotions (Arntz et al., 2019; Kim et al., 2020; Song and Gao, 2020; Verweij et al., 2021). Maternal anxiety is an important risk factor for problem behaviors in preschools. According to previous studies and the results of this study, interventions to reduce maternal anxiety helped reduce preschoolers’ problem behaviors during the COVID-19 epidemic. Most importantly, we found that maternal trait mindfulness acted as a buffer against the impact of maternal anxiety on preschoolers’ problem behaviors. Therefore, when the COVID-19 epidemic is far from over, mothers, as the main caregivers of preschoolers, should try to improve their mindfulness through mindfulness training programs for the healthy development of children; this will help alleviate the adverse effects of the COVID-19 epidemic and self-anxiety on preschoolers’ problem behaviors.

### Limitations and future research

Although the current study examined the risk and protective factors for preschoolers’ problem behaviors in the special context of the COVID-19 epidemic, several limitations of this study are worth mentioning. First, we used a cross-sectional study design during the COVID-19 epidemic; therefore, it is unclear whether the epidemic will continue to affect preschoolers’ problem behaviors after the COVID-19 epidemic ends in China. Second, the participants were recruited only from China; therefore, the findings might not be generalizable to populations from other countries during the COVID-19 epidemic. This study was conducted during the COVID-19 epidemic, so there may be a potential historical effect. Finally, the current study recruited participants via convenient cluster sampling from a middle school in Southeast China via an online survey that was conducted during the period of repeated rebound of the epidemic in China. In-depth family background information of participants was not collected. Therefore, future studies could examine in greater depth the different influences of other family characteristics on this research question, such as urban and rural mothers, and single and non-single mothers. In addition, we did not measure fathers’ work–family conflict and other psychological variables, which could be explored in depth in future research based on family systems theory.

## Conclusion

WFC was positively associated with preschoolers’ problem behaviors, and maternal anxiety was an important mediator of this association. So, WFC could cause maternal anxiety and lead to more problematic behaviors in children. Besides, maternal anxiety was positively associated with preschoolers’ problem behaviors, and trait mindfulness was an important moderator of this association.

## Data availability statement

The raw data supporting the conclusions of this article will be made available by the authors, without undue reservation.

## Ethics statement

The studies involving humans were approved by Institutional Review Board of Kunsan National University. The studies were conducted in accordance with the local legislation and institutional requirements. The participants’ parents/guardians provided their written informed consent to participate in this study.

## Author contributions

XJ: Conceptualization, Data curation, Methodology, Writing – original draft. JA: Investigation, Supervision, Writing – review & editing.
